# Accelerating High-Entropy Alloy Design via Machine Learning: Predicting Yield Strength from Composition

**DOI:** 10.3390/ma19010196

**Published:** 2026-01-05

**Authors:** Seungtae Lee, Seok Su Sohn, Hae-Seok Lee, Donghwan Kim, Yoonmook Kang

**Affiliations:** 1 Department of Materials Science and Engineering, Korea University, Seoul 02841, Republic of Korea; tmdxo12344@korea.ac.kr (S.L.); sssohn@korea.ac.kr (S.S.S.); donghwan@korea.ac.kr (D.K.); 2 Graduate School of Energy and Environment (KU-KIST Green School), Korea University, Seoul 02841, Republic of Korea; lhseok@korea.ac.kr; 3 Department of Integrative Energy Engineering, Korea University, Seoul 02841, Republic of Korea

**Keywords:** high entropy alloys, machine learning, yield strength prediction, data-driven modeling, alloy design

## Abstract

**Highlights:**

**What are the main findings?**

- Developed a machine learning model predicting HEA yield strength from composition.
- Gradient Boosting achieved the best performance with an R^2^ of 0.85.
- Model captures experimental yield-strength trends across diverse HEA categories.

**What are the implications of the main findings?**

- Enables fast screening of HEA compositions with targeted high yield strength.
- Reduces trial-and-error experiments, saving resources and energy in HEA design.
- Offers a general framework extendable to other mechanical properties of HEAs.

**Abstract:**

High-entropy alloys (HEAs) have attracted significant attention due to their exceptional physical, chemical, and mechanical properties. The current development of HEAs primarily depends on time-consuming and costly trial-and-error approaches, which not only hinder the efficient exploration of new compositions but also result in unnecessary resource and energy consumption, thereby negatively affecting sustainable development and production. To address this challenge, this study introduces a machine learning-based methodology for predicting the yield strengths of various HEA compositions. The model was trained using 181 data points and achieved an R^2^ performance score of 0.85. To further assess its reliability and generalization capability, the model was validated using external data not included in the collected dataset. The validation was performed across four categories: modified Cantor alloys, refractory HEAs, eutectic HEAs, and other HEAs. The predicted yield strength trends were found to align with the actual experimental trends, demonstrating the model’s robust performance across various categories of HEAs. The proposed machine learning approach is expected to facilitate the combinatorial design of HEAs, thereby enabling efficient optimization of compositions and accelerating the development of novel alloys. Moreover, it has the potential to serve as a guideline for sustainable alloy design and environmentally conscious production in future HEA development.

## 1. Introduction

Metallic materials have historically been integral to the advancement of civilizations, shaping eras such as the Bronze and Iron Ages. Traditionally, these materials have been utilized as alloys, formed by incorporating small quantities of additional elements into base metals to enhance or impart specific properties [1]. However, conventional alloys are constrained by a limited range of possible compositions [2]. Consequently, high-entropy alloys (HEAs) have emerged as promising alternatives, attracting significant attention due to their exceptional physical, chemical, and mechanical properties. HEAs are alloys composed of five or more principal elements in approximately equal proportions (5–35%), maintaining stable solid solutions due to their high configurational entropy. This distinctive composition enables HEAs to offer a significantly broader range of combinations than conventional alloys [3,4,5,6]. Furthermore, certain HEAs have been proposed as potential substitutes for environmentally detrimental alloys or as promising candidates capable of delivering both energy efficiency and environmental sustainability in specific applications [7].

The vast potential for compositional diversity has driven growing research interest in developing HEAs with novel and enhanced properties. However, the current development of HEAs predominantly relies on trial-and-error methods, which, although reliable, are inherently inefficient and costly. From a sustainability perspective, such repetitive experimental approaches can result in unnecessary energy consumption and material waste, emphasizing the need for reliable predictive tools. Consequently, research in metallurgy has increasingly focused on predicting mechanical properties using theoretical models. These models typically integrate classical equations, such as the Hall–Petch equation [8,9] and Vegard’s law [10], to propose predictive formulas [11,12,13,14,15,16,17]. However, the applicability of these formulas is often restricted to specific conditions, rendering them unsuitable for generalized predictions. As a result, predicting mechanical properties and developing new HEAs using theoretical models remain complex and imprecise.

Artificial intelligence (AI) has emerged as a transformative tool across numerous fields, including medicine [18], chemistry and biology (e.g., protein structure prediction) [19,20], autonomous driving [21], robotics [22], and materials science [23,24,25,26,27]. Its application in HEA research has shown significant potential for advancing the field. For instance, Hou et al. [28] proposed a hybrid model for phase prediction in HEAs that integrates support vector machine (SVM), k-nearest neighbor (KNN), decision tree (DT), logistic regression (LR), and random forest (RF) with a conflict-resolution mechanism based on Dempster–Shafer evidence theory. This approach achieved over 83.3% accuracy in distinguishing between various phases, including single-phase solid solution (SS), amorphous phase (AM), intermetallic compound (IM), and combined SS + IM phases. Similarly, Huang et al. [29] explored phase prediction using machine learning, comparing the performance of KNN, SVM, and artificial neural network (ANN) models. Their findings indicated that ANN outperformed the other models, achieving accuracies of 74.3% for ternary classification (SS, IM, and SS + IM) and ≥78.9% for binary classifications. Machine learning has also been employed to predict mechanical properties. He et al. [30] used machine learning to design refractory high-entropy alloys (RHEAs) with high-temperature resistance, achieving R^2^ scores of 0.942 and 0.892 for yield strength and fracture strain predictions, respectively, using the RF model. The trained models facilitated the screening of alloy compositions and led to experimental proposals of alloys exhibiting high yield strength and excellent fracture strain. Similarly, Giles et al. [31] applied machine learning to predict the yield strength of alloys at high temperatures, achieving an R^2^ score of 89.5% with the RF model. They also used a genetic algorithm to identify compositions that maximize yield strength and employed Shapley Additive Explanations (SHAP) analysis to enhance the interpretability of the prediction results. Additionally, Lee et al. [32] conducted a comprehensive comparison of various models, including black-box models (ensemble tree regressor, random forest, and gradient boosting regressor), white-box models (symbolic regressor), and grain boundary sliding (GBS) models. Experimental validation revealed that the symbolic regressor, capable of generating interpretable mathematical expressions rooted in physical principles, achieved superior prediction accuracy compared to the other models.

**Figure 1:**
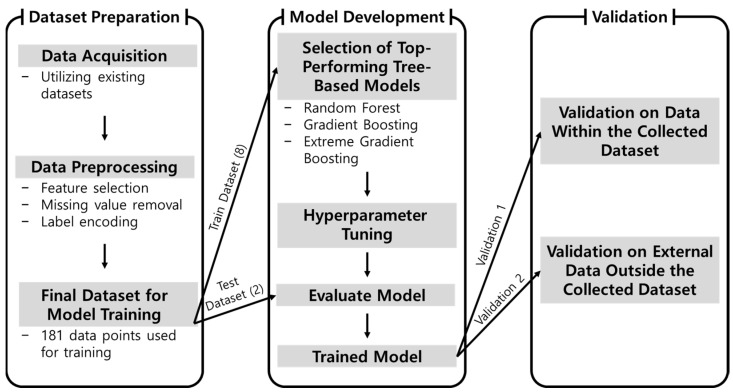
Overview of the machine learning model development process for predicting the yield strength of HEAs based on elemental composition.

Machine learning-based approaches can generate accurate predictions solely from data without relying on explicit physical theories. This capability has substantially advanced research in metallic materials and HEAs, where such methods have been actively applied. Although several studies have attempted to predict yield strength, models specifically designed for diverse HEA systems remain limited, and direct efforts to use elemental composition as input for prediction have not yet been undertaken. Establishing a methodology capable of directly predicting mechanical strength from composition through combinatorial design [33,34] is therefore essential. This approach enables the design of HEAs with targeted mechanical properties and accelerates the discovery of new HEAs. This study addresses this gap by introducing a machine learning methodology for yield strength prediction based on elemental composition. Moreover, the proposed approach is expected to contribute significantly to the future design and development of HEAs that account for both energy efficiency and environmental sustainability. An overview of the proposed methodology is presented in Figure 1.

## 2. Materials and Methods

### 2.1. Data Collection and Preprocessing

The dataset utilized in this study was derived from the work of Borg et al. [35]. The original dataset consisted of 1545 data points. Based on prior domain knowledge, a feature selection process was conducted to identify the features suitable for model training. Data points with missing values in the selected features were subsequently removed, and label encoding was applied where necessary to enable model training. Following this preprocessing procedure, a refined dataset comprising 181 data points was obtained. Detailed descriptions of the features included in the final dataset are provided below.

1. Grain size: Grain size affects key mechanical properties such as yield strength, fracture strength, creep, and ductility [8,9,36,37,38,39]. In this study, grain size values were recorded in micrometers (μm).
2. Processing method: The mechanical properties of a material vary significantly based on its processing method [40,41]. The dataset included processing methods such as casting, wrought processing, annealing, and powder processing, which were label-encoded to facilitate model training.
3. Crystal structure: The crystal structure plays a crucial role in determining mechanical properties. For instance, face-centered cubic (FCC) structures exhibit high ductility and toughness due to the presence of multiple slip systems, whereas body-centered cubic (BCC) structures demonstrate higher strength but lower ductility because of fewer slip systems. In this study, crystal structures were categorized as BCC, FCC, or others, and subsequently label-encoded for model training.
4. Mechanical testing method: Variability in mechanical testing can influence experimental outcomes, making it important to account for this factor in the dataset. In this study, only data obtained from room-temperature tests were included. The test types, tensile and compression, were processed using label encoding.
5. Elemental composition: The atomic percentages (at%) of constituent elements, including Co, Cr, Fe, Mn, Ni, Nb, Ta, Ti, Zr, Al, Hf, W, Mo, V, Cu, and C, were recorded for each HEA.

Temperature is a crucial factor in determining alloy properties. However, in this study, it was assumed that temperature effects could be indirectly inferred from parameters such as grain size and processing method. Consequently, the dataset did not include a separate feature for processing temperature. Furthermore, since only mechanical testing data obtained at room temperature were considered, testing temperature was also excluded as a feature. The complete dataset is provided in the Supplementary Materials.

### 2.2. Model Training and Evaluation

This study employed tree-based machine learning models. Although deep learning has achieved remarkable progress and demonstrates exceptional performance with image and text datasets, tree-based models generally outperform other methods when applied to tabular datasets, particularly those with a limited number of data points [42,43]. Based on this, RF, extreme gradient boosting (XGBoost), and gradient boosting (GB) models were selected for analysis.

The dataset was divided into training and test sets using an 8:2 ratio. Hyperparameter tuning was conducted using GridSearchCV to optimize model performance. The evaluation metrics used to assess model performance included the coefficient of determination (R^2^), root mean square error (RMSE), and mean absolute percentage error (MAPE), defined as follows:

$$R^{2}=1-\frac{\sum_{i=1}^{n}\left(y_{i}-\hat{y_{i}}\right)}{\sum_{i=1}^{n}\left(y_{i}-\bar{y}\right)}$$

$$RMSE=\sqrt{\frac{1}{n}\sum_{i=1}^{n}{\left(y_{i}-\hat{y_{i}}\right)}^{2}}$$

$$MAPE=\frac{1}{n}\sum_{i=1}^{n}\frac{\left|\hat{y_{i}}-y_{i}\right|}{y_{i}}\times 100$$

where n represents the total number of data points, $y_{i}$ denotes the observed value for the ith data point, $\hat{y_{i}}$ represents the corresponding predicted value, and $\bar{y}$ is the mean of all observed values. These metrics were used to identify the model demonstrating the highest prediction accuracy, which was then selected as the final prediction model for HEA yield strength.

## 3. Results

### 3.1. Machine Learning Prediction Results

The results of model training using the RF, XGBoost, and GB models, along with a comparison of their prediction performance on the test dataset, are summarized in Table 1.

**Figure 2:**
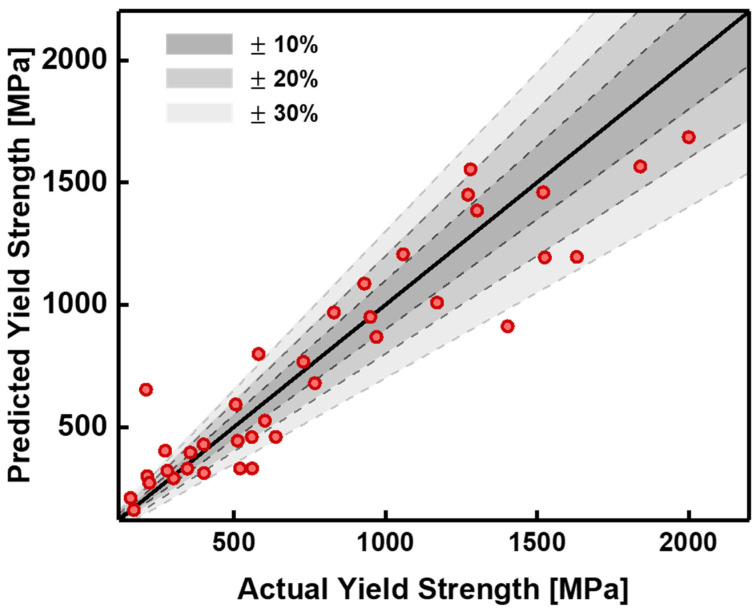
Prediction performance of the GB model, including an error analysis of the test data with boundary lines corresponding to ±10%, ±20%, and ±30% error thresholds. Darker regions indicate higher prediction accuracy.

The GB model demonstrated the highest performance, achieving R^2^, RMSE, and MAPE values of 0.8538, 192.9876 MPa, and 23.62%, respectively. These results indicate that the GB model achieved superior prediction accuracy compared to the other models. The prediction results obtained using the GB model are illustrated in Figure 2 and Figure S1.

Figure S1 illustrates the relationship between the actual and predicted yield strengths, where the x- and y-axes represent the actual and predicted values, respectively. The black reference line (y = x) indicates perfect agreement between predictions and observations, with data points closer to this line signifying higher prediction accuracy. The graph shows the predictions generated by the GB model closely align with the reference line, demonstrating the model’s robust performance. For a more detailed analysis, the data were divided into two ranges: low yield strength (<1000 MPa) and high yield strength (≥1000 MPa). The error rate was used to evaluate prediction accuracy in these ranges and was calculated as:

$$error\;rate=\frac{\hat{y}-y}{y}\times 100$$

where $\hat{y}$ denotes the predicted value and y represents the actual observed value. Unlike MAPE, this calculation retains the sign of the deviation, providing additional insight into the direction of the prediction error.

Figure 2 compares the predicted and actual values from the test dataset by visualizing error rates across the two yield strength ranges. The graph includes boundaries representing error thresholds of ±10%, ±20%, and ±30%, with color-coded regions that enable easy identification of error rates for individual data points. The darkest region corresponds to error rates within ±10%, while progressively lighter shades represent ±20% and ±30% error ranges. The comparison revealed that, for the low-yield-strength range, 23.1% of data points had error rates within ±10%, 61.5% within ±20%, and 73.1% within ±30%. For the high-yield-strength range, 18.1% of data points were within ±10%, 63.6% within ±20%, and 90.9% within ±30%. While the proportions within the ±10% and ±20% thresholds were comparable across both ranges, examining the ±30% error threshold shows that the high-yield-strength range contains a larger proportion of data points within this margin. This indicates that the predictions for the high-yield-strength range are relatively more accurate than those for the low-yield-strength range. These findings demonstrate the strong performance of the GB model in predicting high-yield-strength data. A detailed analysis of these results is provided in Figure S2.

Although the overall prediction accuracy of the proposed model is moderate compared to that reported in other machine learning studies, its enhanced performance in the high-yield-strength range is particularly advantageous for predicting HEA compositions with superior mechanical strength. To assess the reliability of the model, two validation approaches were employed: (1) validation using data included in the collected dataset and (2) validation using external data not included in the collected dataset.

### 3.2. Model Application to the Collected Dataset

**Figure 3:**
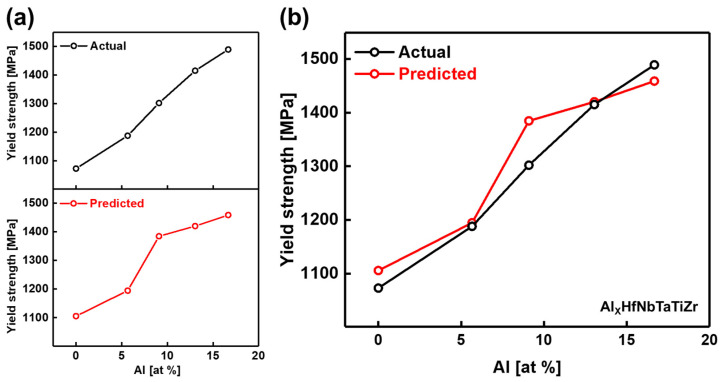
Comparison of actual yield strength data (black) and predicted results (red) obtained from the trained GB model, based on data from Lin et al. (**a**) Graph showing actual and predicted yield strength values. (**b**) Combined graph illustrating a direct comparison between actual and predicted values.

Predictions were made using data randomly selected from the collected dataset to evaluate the model’s performance. In particular, experimental data on Al_x_HfNbTaTiZr HEAs (x = 0.0, 0.3, 0.5, 0.75, and 1.0) reported by Lin et al. [44] were used. In that study, the mechanical properties of HEAs were examined by varying the atomic ratio of Al. The compositions and experimental conditions of these alloys were input into the trained GB model, and the predicted yield strengths were compared with the experimentally measured values. The results are shown in Figure 3a.

This study posits that the addition of Al induces lattice distortion due to differences in atomic radii, leading to a solid-solution hardening effect. Furthermore, strong bonding interactions are attributed to the formation of p–d hybridized orbitals between Al and transition metals. Consequently, the inclusion of Al is concluded to enhance yield strength. The trained model successfully captured this trend without prior knowledge of the underlying physical mechanisms, achieving high prediction accuracy with a MAPE of 2.44% (see Figure 3b).

### 3.3. Model Application to Data Outside the Collected Dataset

**Figure 4:**
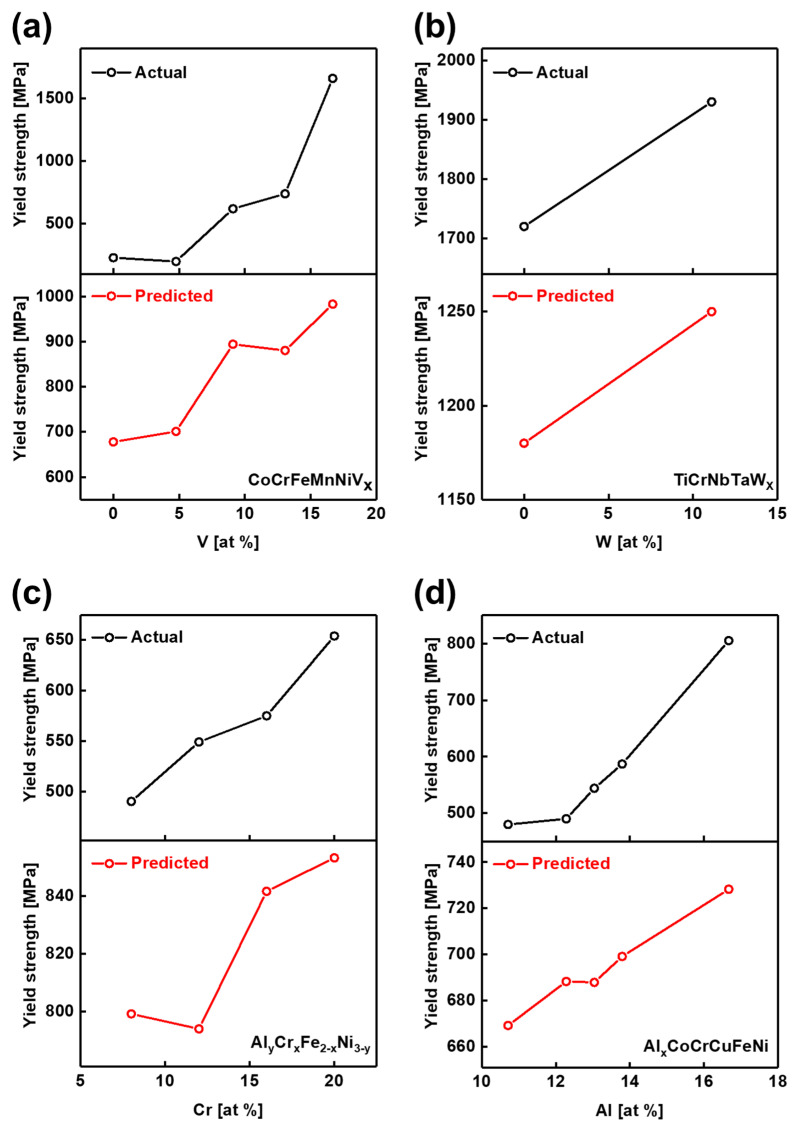
Validation of the GB model predictions against actual yield strength values reported in external literature. (**a**) Comparison of actual and predicted yield strength values for vanadium-containing modified Cantor alloys. (**b**) Comparison of actual and predicted values for RHEAs. (**c**) Comparison of actual and predicted values for EHEAs. (**d**) Comparison of actual and predicted values for other high-entropy alloys.

External data not included in the collected dataset were used for validation to evaluate the reliability and generalization capability of the model. These external data were obtained from various HEAs categories, including modified Cantor alloys, RHEAs, eutectic high-entropy alloys (EHEAs), and other HEAs. The predicted yield strength values for each category were compared with experimental results reported in the literature, with the overall results presented in Figure 4.

#### 3.3.1. Vanadium-Containing Modified Cantor Alloys

The Cantor alloy, primarily composed of CoCrFeMnNi, is known for its FCC structure [4,45]. Recent efforts to enhance its mechanical properties have focused on alloying with additional elements such as Al [46,47], V [48], and Ti [49,50]. For model validation, data on the microstructure and mechanical properties of CoCrFeMnNiV_x_ (x = 0.0, 0.25, 0.5, 0.75, and 1.0) HEAs reported by Stepanov et al. [48] were randomly selected. This dataset was appropriate because it contained all the experimental conditions and compositional details required by the model. The experimentally reported yield strength values for increasing V content were 230, 200, 620, 740, and 1660 MPa, whereas the GB model predicted 678, 701, 894, 880, and 983 MPa, respectively. Although the absolute values differed, rescaling the graphs for comparison revealed that both the experimental and predicted results exhibited a consistent increasing trend in yield strength with higher V content, forming a similar W-shaped pattern (Figure 4a).

#### 3.3.2. Refractory High Entropy Alloys

RHEAs are HEAs composed of high-temperature-resistant elements such as Ti, Mo, Ta, and W. These alloys, which predominantly exhibit a BCC structure, are widely recognized for their exceptional mechanical properties at elevated temperatures, making them highly suitable for high-temperature applications [4,6,51,52]. Among various studies, data from You et al. [53] were randomly selected for model validation. This study investigated the mechanical properties of TiCrNbTaWx (x = 0.0, 0.5) RHEAs, with a particular focus on wear behavior. The reported yield strength values were 1.72 GPa and 1.93 GPa for x = 0.0 and x = 0.5, respectively, whereas the GB model predicted 1.18 GPa and 1.25 GPa. Despite differences in absolute values, the model successfully captured the increasing trend in yield strength with higher W content Figure 4b clearly illustrates this trend, showing consistent behavior between experimental and predicted results when plotted on adjusted scales.

#### 3.3.3. Eutectic High Entropy Alloys

EHEAs leverage the eutectic solidification concept, wherein two or more solid phases crystallize simultaneously from the liquid phase, forming a dual-phase structure that offers a favorable balance between strength and ductility [1,54]. For model validation within the EHEAs category, data from Mao et al. [55] on Al_y_Cr_x_Fe_2-x_Ni_3-y_ alloys were randomly selected from available studies. In that work, the Cr concentration (x) was systematically varied, while the Fe content was adjusted to maintain a consistent 3:2 ratio between (Al + Ni) and (Cr + Fe). Additionally, the y value was fine-tuned to preserve a constant valence electron concentration (VEC), thereby facilitating eutectic alloy formation. The experimental results showed that the actual yield strength increased to 490, 549, 575, and 654 MPa as the Cr concentration (x) increased to 0.2, 0.4, 0.8, and 1.0, respectively. The machine learning model predicted yield strengths of 799, 794, 842, and 853 MPa for the same compositions. When plotted on adjusted graph scales (see Figure 4c), the predicted yield strength values closely followed the experimental trend, with only a slight deviation observed at x = 0.4 (Cr = 12 at%).

#### 3.3.4. Other High Entropy Alloys (AlxCoCrCuFeNi)

HEAs encompass a wide range of compositions and structural configurations. Among these, data on Al_x_CoCrCuFeNi HEAs reported by Mahato et al. [56] were randomly selected for model validation. The study found that as the Al composition (x) increased incrementally from 0.6, 0.7, 0.75, and 0.8 to 1.0, the alloy underwent a structural transition from an FCC to a BCC phase. This phase evolution was accompanied by yield strength values of 480, 490, 544, 587, and 805 MPa, respectively. The machine learning model predicted corresponding yield strengths of 669, 688, 688, 699, and 728 MPa. Although slight deviations in slope were observed around x = 0.7 (Al = 12.28%) (see Figure 4d), the overall trend of increasing yield strength with higher Al content was accurately captured.

## 4. Discussion

The results presented in Figure 3 show a consistent alignment between the experimental and predicted trends. However, discrepancies arise when comparing yield strength values directly, which can be attributed to three key factors:

1. Deviations due to experimental process conditions: The applied machine learning model was developed using a simplified framework that did not account for detailed experimental processing parameters. Since yield strength is highly sensitive to specific process conditions, the absence of such information in the input data can lead to discrepancies between the predicted and experimentally measured yield strength values.
2. Variation induced by environmental factors: Even under identical processing conditions, differences in experimental setups, equipment calibration, or testing environments can cause fluctuations in yield strength. Such environmental variations introduce additional uncertainty, further complicating the accurate prediction of yield strength values.
3. Insufficient data availability: The performance of the machine learning model strongly depends on the quality and comprehensiveness of the input data. Limited data availability adversely affects prediction accuracy and amplifies the uncertainty arising from process conditions and environmental variations, reducing the model’s ability to produce generalized and reliable predictions. For instance, as shown in Figure S3, data points within the 12–13 at% range accounted for only 1.33% of the total dataset, and even when this range was expanded to 10–15 at%, the data represented only 4.11%. This scarcity of data, particularly near 12 at%, explains the prediction error observed in this region and underscores the challenges posed by data insufficiency in achieving accurate yield strength predictions.

However, the proposed methodology effectively captured the underlying trends across diverse HEA systems, even when trained on a limited dataset. Incorporating additional data and conducting further model training are expected to enhance its capability to predict both trends and precise yield strength values (see Section 3.2). The ability to accurately identify trends, even in the absence of exact yield strength predictions, holds significant potential for the exploration of novel materials and the design of alloys with tailored mechanical properties.

Furthermore, the methodology proposed in this study can be extended in several directions through future research.

1. Prediction of additional mechanical properties: In practical industrial applications, material selection is typically based on the comparison of multiple mechanical properties rather than a single property. However, in the present study, the analysis was intentionally limited to yield strength. By retaining the same methodology and replacing the target output from yield strength with other mechanical properties, such as ductility and ultimate tensile strength, the proposed framework can be extended to predict these properties. Such an extension to additional mechanical properties would provide more practical guidance for the industrial application of HEAs.
2. Expansion of input features: Although this study primarily focused on composition-based prediction, the mechanical behavior of HEAs in practice is influenced not only by elemental composition but also by multiple interacting factors. These include microstructural morphology and its distribution across multiple length scales, grain size, grain boundary distribution, dislocation density, and detailed processing conditions, all of which are well known to have a direct influence on yield strength. Accordingly, incorporating microstructural and processing-related information is expected to further improve prediction accuracy. Such information may be obtained either through direct experimentation or manual data collection from the literature; alternatively, it can be more efficiently acquired by leveraging natural language processing tools, such as ChemDataExtractor [57,58,59], to automatically extract relevant data from existing studies.
3. Experimental validation for model reliability: In this study, model validation was conducted using literature-based data. However, to verify that the proposed model operates reliably in practice, validation should extend to include experimental verification. Such experimental validation would be an important step toward demonstrating the proposed approach to real-world alloy design and industrial environments.

**Figure 5:**
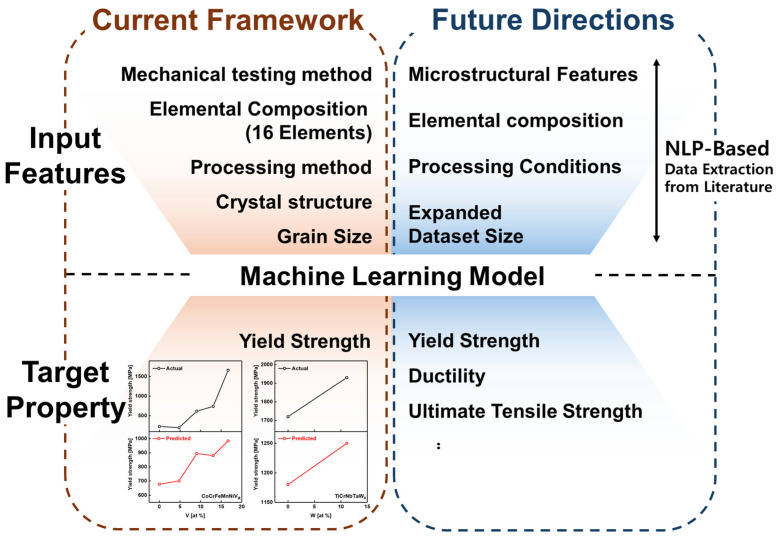
Schematic overview of the current machine learning framework and its future directions.

To summarize the present study and its prospective extensions, a schematic overview of the proposed framework and future research directions is provided in Figure 5.

## 5. Conclusions

This study proposed a machine learning-based methodology for predicting the yield strength of HEAs. A total of 181 data points, selectively extracted and preprocessed from the dataset provided by Borg et al., were used to train the model. Among the tree-based algorithms evaluated—RF, GB, and XGBoost—the GB model exhibited the highest performance, achieving an R^2^, RMSE, and MAPE of 0.8538, 192.9876 MPa, and 23.62%, respectively.

Two validation approaches were employed to evaluate the model’s performance: (1) validation using data within the collected dataset and (2) validation using external data not included in the collected dataset. Validation with the collected dataset showed strong agreement between the predicted and experimental yield strength values, achieving a MAPE of 2.44% and accurately reproducing the observed trends. External data validation demonstrated that the model successfully captured yield strength trends across diverse HEA categories, including modified Cantor alloys, RHEAs, EHEAs, and other HEA compositions.

The proposed model effectively identified yield strength trends across a broad range of HEAs, even when trained on a limited dataset. Its predictive accuracy is expected to improve with the inclusion of additional data and further model training. The ability to capture trends, even in the absence of exact yield strength predictions, can help minimize redundant experimental efforts, reduce resource consumption, and provide valuable insights for the discovery and combinatorial design of HEAs with tailored mechanical properties. Furthermore, the proposed methodology can be extended to other mechanical properties, such as ductility and ultimate tensile strength, offering a robust framework to accelerate the exploration and development of HEAs with diverse mechanical characteristics. These advancements are anticipated to contribute significantly to sustainable alloy design, development, and production.

## Figures and Tables

**Table 1:** Performance comparison of tree-based machine learning models on the test dataset for yield strength prediction.

Model	R2	RMSE [MPa]	MAPE [%]
RF	0.7865	233.2481	28.88
XGBoost	0.8173	215.7462	27.06
GB	0.8538	192.9876	23.62

## Data Availability

The original data presented in the study are openly available at https://github.com/KU-Seungtae/HEAs_ML accessed on 24 November 2025.

## Supplementary Materials

The following supporting information can be downloaded at: https://www.mdpi.com/article/10.3390/ma19010196/s1, Figure S1. Prediction performance of the GB model, showing a comparison between the actual and predicted yield strength values for the training (light red) and test (dark red) datasets. The black line (y = x) indicates perfect prediction. Figure S2. Histogram illustrating the error rate distribution of test set predictions, categorized based on yield strength values greater or less than 1000 MPa. Figure S3. Histogram depicting the distribution of elemental composition data points across specific intervals (excluding the composition value of 0 at%). (**a**) Distribution with a composition interval of 2 at%. (**b**) Distribution with a composition interval of 5 at%. Figure S4. Yield strength prediction results obtained using XGBoost trained with 5-fold cross-validation and TPE-based hyperparameter optimization, evaluated across four representative alloy categories. Table S1. Results of hyperparameter tuning for each model.

## Abbreviations

The following abbreviations are used in this manuscript:

AI	Artificial intelligence
AM	Amorphous phase
ANN	Artificial neural network
BCC	Body-centered cubic
DT	Decision tree
EHEAs	Eutectic high-entropy alloys
FCC	Face-centered cubic
GB	Gradient boosting
GBS	Grain boundary sliding
HEAs	High-entropy alloys
IM	Intermetallic compound
KNN	K-nearest neighbor
LR	Logistic regression
MAPE	Mean absolute percentage error
RF	Random forest
RHEAs	Refractory high-entropy alloys
RMSE	Root mean square error
R^2^	Coefficient of determination
SHAP	Shapley Additive Explanations
SS	Solid solution
SVM	Support vector machine
XGBoost	Extreme gradient boosting
