# Supplementary material for: Accelerating High-Entropy Alloy Design via Machine Learning: Predicting Yield Strength from Composition
Source: Materials (Basel). 2026 Jan 5;19(1):196. doi: 10.3390/ma19010196 (PMC12786949; doi:10.3390/ma19010196)
Supplement: Supplementary file 1 [file materials-19-00196-s001.zip › materials-4036213-supplementary.pdf]

materials

MDPI

Supplementary Materials

Accelerating High-Entropy Alloy Design via Machine Learning: Predicting Yield Strength from Composition

Seungtae Lee [1], Seok Su Sohn [1], Hae-Seok Lee [2,3], Donghwan Kim [1] and Yoonmook Kang [2,3,*]

[1] Department of Materials Science and Engineering, Korea University, Seoul 02841, Republic of Korea; tmdxo12344@korea.ac.kr (S.L.); sssohn@korea.ac.kr (S. S. Sohn); donghwan@korea.ac.kr (D.K.)
[2] Graduate School of Energy and Environment (KU-KIST Green School), Korea University, Seoul 02841, Republic of Korea; lhseok@korea.ac.kr (H.-S.L.); ddang@korea.ac.kr (Y.K.)
[3] Department of Integrative Energy Engineering, Korea University, Seoul 02841, Republic of Korea; lhseok@korea.ac.kr (H.-S.L.); ddang@korea.ac.kr (Y.K.)
* Correspondence: ddang@korea.ac.kr; Tel.: +82-2-3290-3713

Table S1. Results of hyperparameter tuning for each model.

ML model	Hyperparameter	Value	Prediction results
RF	bootstrap	True	$R^2 = 0.79$ RMSE = 233.25 MPa
	max_depth	20	
	max_features	0.8	
	min_samples_leaf	1	
	min_samples_split	2	
	n_estimators	100	
GB	learning_rate	0.05	$R^2 = 0.85$ RMSE = 192.99 MPa
	max_depth	5	
	max_features	sqrt	
	min_samples_leaf	1	
	min_samples_split	2	
	n_estimators	200	
	subsample	0.8	
XGBoost	colsample_bytree	1	$R^2 = 0.85$ RMSE = 192.99 MPa
	gamma	0	
	learning_rate	0.1	
	max_depth	5	
	min_child_weight	1	
	n_estimators	100	
	reg_alpha	1	
	reg_lambda	3	
	subsample	1	

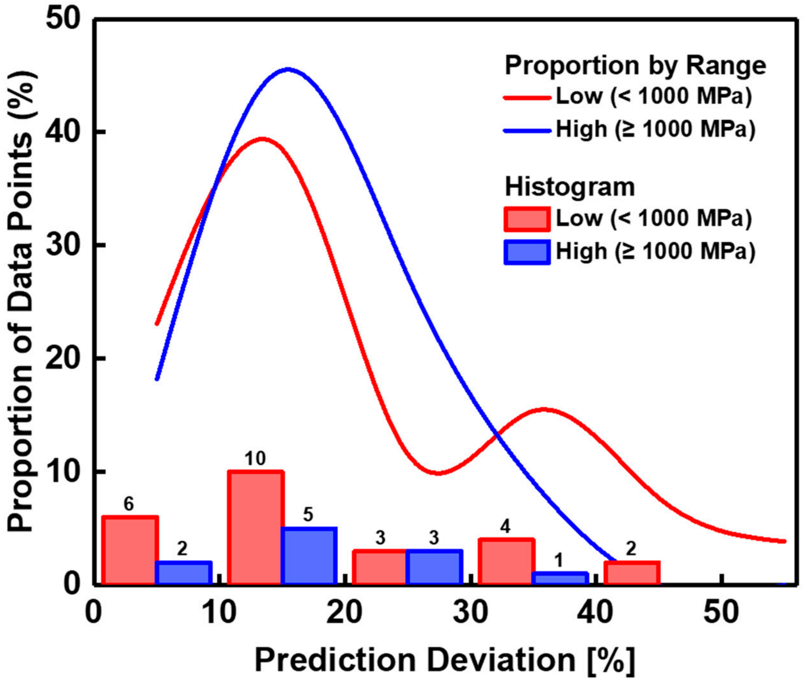

Figure S1. Histogram illustrating the error rate distribution of test set predictions, categorized based on yield strength values greater or less than 1000 MPa.

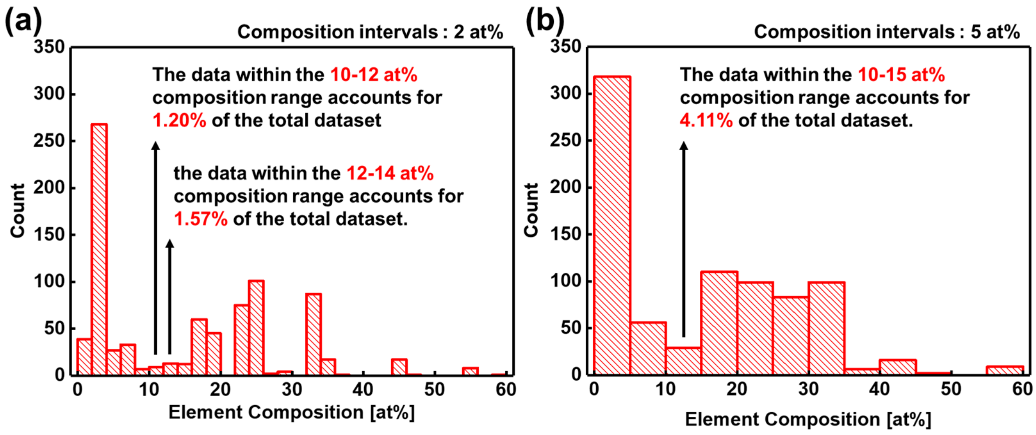

Figure S2. Histogram depicting the distribution of elemental composition data points across specific intervals (excluding the composition value of 0 at%). (**a**) Distribution with a composition interval of 2 at%. (**b**) Distribution with a composition interval of 5 at%.

Prediction Code (RF, GB, XGBoost).

RF

```
#%%
# Load Dataset and Split Data
# Import necessary libraries
import pandas as pd
from sklearn.model_selection import train_test_split, GridSearchCV
from sklearn.ensemble import RandomForestRegressor
from sklearn.metrics import mean_squared_error, r2_score
import numpy as np
```

```
# 1. Load the Excel dataset
file_path = '/home/korea/works/231214_MPEA_dataset_remove feature.xlsx'
data = pd.read_excel(file_path)

# 2. Define input (X) and output (Y) columns
X = data.iloc[:, 0:20]  # Columns A to V
Y = data.iloc[:, 20]    # Column W (YS values)

# 3. Split data into training (80%) and testing (20%) sets
X_train, X_test, Y_train, Y_test = train_test_split(X, Y, test_size=0.2, ran-
dom_state=123)

#%%
# First Stage: Initial Grid Search
# Define initial hyperparameter grid
param_grid = {
    'n_estimators': [100, 200, 300, 500, 700],                # Number of trees
    'max_depth': [10, 20, 30, 50, 70, None],                  # Maximum depth of
trees
    'min_samples_split': [2, 5, 10, 15],                      # Minimum samples
required to split a node
    'min_samples_leaf': [1, 2, 4, 6, 8],                      # Minimum samples
required at leaf node
    'max_features': ['auto', 'sqrt', 'log2', 0.2, 0.5, 0.8], # Maximum features
considered
    'bootstrap': [True, False]                                # Bootstrap sampling
}

# Perform initial GridSearchCV
rf_model = RandomForestRegressor(random_state=123)
grid_search = GridSearchCV(estimator=rf_model, param_grid=param_grid, cv=3,
n_jobs=-1, verbose=2, scoring='r2')
grid_search.fit(X_train, Y_train)

# Extract and display best parameters from the first stage
best_params_initial = grid_search.best_params_
print(f"Initial Best Parameters: {best_params_initial}")

#%%
# Second Stage: Refined Grid Search
# Define refined hyperparameter grid
param_grid_refined = {
```

```
    'n_estimators': [90, 100, 110, 120, 130, 150, 180],       # Number of trees 
(refined)
    'max_depth': [15, 18, 20, 22, 25, 28, 30],                # Maximum depth of 
trees (refined)
    'min_samples_split': [2, 3, 4, 5, 6],                     # Minimum samples re-
quired to split a node (refined)
    'min_samples_leaf': [1, 2, 3],                            # Minimum samples re-
quired at leaf node (refined)
    'max_features': [0.6, 0.7, 0.75, 0.8, 0.85, 0.9],        # Maximum features 
considered (refined)
    'bootstrap': [True]                                       # Bootstrap sampling 
(fixed)
}

# Perform refined GridSearchCV
grid_search_refined = GridSearchCV(estimator=rf_model, param_grid=param_grid_re-
fined, cv=3, n_jobs=-1, verbose=2, scoring='r2')
grid_search_refined.fit(X_train, Y_train)

# Evaluate the best model from the refined search
best_rf_model_refined = grid_search_refined.best_estimator_
Y_pred_best_refined = best_rf_model_refined.predict(X_test)

#%%
# Evaluate Metrics and Feature Importance
# Calculate evaluation metrics
best_mse_refined = mean_squared_error(Y_test, Y_pred_best_refined)
best_rmse_refined = best_mse_refined ** 0.5
best_r2_refined = r2_score(Y_test, Y_pred_best_refined)

print(f"Tuned Model (Refined) RMSE: {best_rmse_refined:.2f}")
print(f"Tuned Model (Refined) R²: {best_r2_refined:.2f}")
print(f"Best Parameters (Refined): {grid_search_refined.best_params_}")
```

GB

```
#%%
# Load Dataset and Split Data
# Import necessary libraries
import pandas as pd
from sklearn.model_selection import train_test_split, GridSearchCV
from sklearn.ensemble import GradientBoostingRegressor
from sklearn.metrics import mean_squared_error, r2_score

# 1. Load the Excel dataset
```

```
file_path = '/home/korea/works/231214_MPEA_dataset_remove feature.xlsx'
data = pd.read_excel(file_path)

# 2. Define input (X) and output (Y) columns
X = data.iloc[:, 0:20]  # Columns A to V
Y = data.iloc[:, 20]    # Column W (YS values)

# 3. Split data into training (80%) and testing (20%) sets
X_train, X_test, Y_train, Y_test = train_test_split(X, Y, test_size=0.2, ran-
dom_state=123)

#%%
# First Stage: Initial Grid Search
# Define initial hyperparameter grid
param_grid = {
    'n_estimators': [100, 200, 300, 500],              # Number of trees
    'learning_rate': [0.01, 0.05, 0.1, 0.2],           # Learning rate
    'max_depth': [3, 4, 5, 6],                         # Maximum depth of trees
    'min_samples_split': [2, 5, 10, 15],               # Minimum samples required
to split a node
    'min_samples_leaf': [1, 2, 4, 6],                  # Minimum samples required
at leaf node
    'subsample': [0.7, 0.8, 0.9, 1.0],                 # Subsampling ratio
    'max_features': ['auto', 'sqrt', 'log2']            # Maximum features used in
trees
}

# Perform initial GridSearchCV
gb_model = GradientBoostingRegressor(random_state=123)
initial_grid_search = GridSearchCV(estimator=gb_model, param_grid=param_grid,
cv=3, n_jobs=-1, verbose=2, scoring='r2')
initial_grid_search.fit(X_train, Y_train)

# Extract and display best parameters from the first stage
best_params_initial = initial_grid_search.best_params_
print(f"Initial Best Parameters: {best_params_initial}")

#%%
# Second Stage: Refined Grid Search
# Use a predefined refined hyperparameter grid
param_grid_refined = {
    'n_estimators': [180, 200, 220, 250],                # Number of trees
    'learning_rate': [0.03, 0.04, 0.05, 0.06, 0.07],     # Learning rate
    'max_depth': [4, 5, 6],                              # Maximum depth of trees
```

```
    'min_samples_split': [2, 3, 4],                     # Minimum samples re-
quired to split a node
    'min_samples_leaf': [1, 2, 3],                      # Minimum samples re-
quired at leaf node
    'subsample': [0.75, 0.8, 0.85, 0.9],                # Subsampling ratio
    'max_features': ['sqrt', 'log2', 0.5, 0.7]          # Maximum features used
in trees
}

# Perform refined GridSearchCV
refined_grid_search = GridSearchCV(estimator=gb_model, param_grid=param_grid_re-
fined, cv=3, n_jobs=-1, verbose=2, scoring='r2')
refined_grid_search.fit(X_train, Y_train)

# Evaluate the best model from the refined search
best_gb_model = refined_grid_search.best_estimator_
Y_pred_best = best_gb_model.predict(X_test)

# Calculate and display evaluation metrics
best_mse = mean_squared_error(Y_test, Y_pred_best)
best_rmse = best_mse ** 0.5
best_r2 = r2_score(Y_test, Y_pred_best)

print(f"Refined Best Parameters: {refined_grid_search.best_params_}")
print(f"Tuned Gradient Boosting Model RMSE: {best_rmse:.2f}")
print(f"Tuned Gradient Boosting Model R²: {best_r2:.2f}")
```

XGBoost

```
#%%
# Load Dataset and Split Data
# Import necessary libraries
import pandas as pd
from sklearn.model_selection import train_test_split, GridSearchCV
from sklearn.metrics import mean_squared_error, r2_score
from xgboost import XGBRegressor
import numpy as np

# 1. Load the Excel dataset
file_path = '/home/korea/works/231214_MPEA_dataset_remove feature.xlsx'
data = pd.read_excel(file_path)

# 2. Define input (X) and output (Y) columns
```

```
X = data.iloc[:, 0:20]  # Columns A to V
Y = data.iloc[:, 20]    # Column W (YS values)

# 3. Split data into training (80%) and testing (20%) sets
X_train, X_test, Y_train, Y_test = train_test_split(X, Y, test_size=0.2, ran-
dom_state=123)

#%%
# First Stage: Initial Grid Search
# Define initial hyperparameter grid
param_grid = {
    'n_estimators': [100, 200, 300, 500],                  # Number of trees
    'learning_rate': [0.01, 0.05, 0.1, 0.2],               # Learning rate
    'max_depth': [3, 4, 5, 6, 8],                          # Maximum depth of
trees
    'min_child_weight': [1, 3, 5, 7],                      # Minimum child weight
    'subsample': [0.6, 0.7, 0.8, 0.9, 1.0],                # Subsample ratio of
training instances
    'colsample_bytree': [0.6, 0.7, 0.8, 0.9, 1.0],         # Subsample ratio of
columns
    'gamma': [0, 0.1, 0.2, 0.3],                           # Minimum loss reduc-
tion for partitioning
    'reg_alpha': [0, 0.01, 0.1, 1],                        # L1 regularization
    'reg_lambda': [1, 1.5, 2, 3]                           # L2 regularization
}

# Perform initial GridSearchCV
xgb_model = XGBRegressor(objective='reg:squarederror', random_state=123)
grid_search = GridSearchCV(estimator=xgb_model, param_grid=param_grid, cv=3,
n_jobs=-1, verbose=2, scoring='r2')
grid_search.fit(X_train, Y_train)

# Extract and display best parameters from the first stage
best_params_initial = grid_search.best_params_
print(f"Initial Best Parameters for XGBoost: {best_params_initial}")

#%%
# Second Stage: Refined Grid Search
# Define refined hyperparameter grid
param_grid_refined = {
    'n_estimators': [90, 100, 120, 150],                    # Number of trees (re-
fined)
    'learning_rate': [0.05, 0.08, 0.1, 0.12],               # Learning rate (re-
fined)
```

```
    'max_depth': [4, 5, 6],                                # Maximum depth of
trees (refined)
    'min_child_weight': [1, 2, 3],                         # Minimum child weight
(refined)
    'gamma': [0, 0.1, 0.2],                                # Gamma value (re-
fined)
    'subsample': [0.8, 0.9, 1.0],                          # Subsample ratio (re-
fined)
    'colsample_bytree': [0.8, 0.9, 1.0],                   # Subsample ratio of
columns (refined)
    'reg_alpha': [0.5, 1, 1.5],                            # L1 regularization
(refined)
    'reg_lambda': [2, 3, 4]                                # L2 regularization
(refined)
}

# Perform refined GridSearchCV
grid_search_refined = GridSearchCV(estimator=xgb_model, param_grid=param_grid_re-
fined, cv=3, n_jobs=-1, verbose=2, scoring='r2')
grid_search_refined.fit(X_train, Y_train)

# Evaluate the best model from the refined search
best_xgb_model = grid_search_refined.best_estimator_
Y_pred_best = best_xgb_model.predict(X_test)

#%%
# Evaluate Metrics
# Calculate evaluation metrics
best_mse = mean_squared_error(Y_test, Y_pred_best)
best_rmse = best_mse ** 0.5
best_r2 = r2_score(Y_test, Y_pred_best)

print(f"Tuned XGBoost Model RMSE: {best_rmse:.2f}")
print(f"Tuned XGBoost Model R²: {best_r2:.2f}")
print(f"Best Parameters (Refined): {grid_search_refined.best_params_}")
```

22
